# Faure’s new social contract fifty years later: Promises and evolutions

**DOI:** 10.1007/s11159-022-09974-6

**Published:** 2022-11-05

**Authors:** Rita Locatelli

**Affiliations:** Università Cattolica del Sacro Cuore, Milan, Italy

**Keywords:** Faure report, new social contract, Futures of Education, common good, shared governance, togetherness

## Abstract

The International Commission on the Development of Education set up by UNESCO in 1971 was chaired by Edgar Faure. The conceptualisation of a new social contract in his work between the 1960s and 1970s had a strong influence on the final report prepared by this commission. Published in 1972, *Learning to be: The world of education today and tomorrow* is commonly known as the Faure report. Although not explicitly mentioned in the report, the idea of a new social contract provided a political framework for re-establishing the particular relationship between education and society, based on a strong belief in an educational democracy which considered citizens as real agents of change. Fifty years after the publication of the Faure report, another report commissioned by UNESCO, on the Futures of Education, has taken up the idea of the social contract, conceiving it as a means to transform education to harness greater cooperation towards more sustainable futures. However, while the understanding of the social contract elaborated by Faure translated into a clear vision of the emancipatory function of education for the fulfilment of individuals within democratic societies, the political discussion on the relationship among the institutions that should govern the new social contract for education presented in the *Futures of Education* report appears less explicit. This article discusses the extent to which the principles underpinning the new social contract for education, especially the notion of education as a common good, provide the political framing of a new social contract *for education*. It examines the relevance of the political discussion of the relationship between education and society elaborated in the Faure report fifty years ago with regard to the formulation of a new social contract for education.

## Résumé

Le nouveau contrat social de Faure cinquante ans après : promesses et évolutions – La Commission internationale sur le développement de l’éducation créée en 1971 par l’UNESCO était présidée par Edgar Faure. La conceptualisation d’un nouveau contrat social dans le cadre de son travail entre les années 60 et 70 a exercé une forte influence sur le rapport final préparé par cette commission. Publié en 1972 sous le titre *Apprendre à être*, il est mieux connu sous le nom de rapport Faure. Bien qu’il n’y soit pas explicitement fait état, l’idée d’un nouveau contrat social apportait un cadre politique permettant de renouer le lien particulier entre l’éducation et la société reposant sur la foi profonde dans une démocratie de l’enseignement dans laquelle les citoyens étaient considérés comme de véritables agents du changement. Cinquante ans après la publication du rapport Faure, un autre rapport commandé par l’UNESCO sur les futurs de l’éducation a repris l’idée du contrat social, voyant en lui un moyen de transformer l’éducation afin d’exploiter une coopération plus vaste en vue d’un avenir plus durable. Néanmoins, tandis que la notion de contrat social élaborée par Faure s’était traduite par une vision claire de la fonction émancipatrice de l’éducation pour l’épanouissement de l’individu au sein des sociétés démocratiques, dans le rapport sur les *futurs de l’éducation*, le débat politique sur le lien entre les institutions auxquelles devrait échoir la direction du nouveau contrat social pour l’éducation apparaît moins explicite. Cet article examine dans quelle mesure les principes sur lesquels reposent le nouveau contrat social pour l’éducation, en particulier la notion d’éducation en tant que bien commun, fournissent le cadre politique d’un nouveau contrat social *pour l’éducation*. Il étudie la pertinence du débat politique sur la relation entre l’éducation et la société, définie il y a cinquante ans dans le rapport Faure, pour la formulation d’un nouveau contrat social pour l’éducation.

## Introduction

Historically, the idea of a “social contract” emerged in the 17th century in the work of Thomas Hobbes (1588–1679) and John Locke (1632–1704) in England, and Jean-Jacques Rousseau (1712–1778) in France. Political and social priorities at the time were focused on the need to ensure the protection of the freedom and rights of citizens. The social contract, or social pact, was therefore considered at the basis of the birth of liberal democracies, whereby the state of nature was replaced, and political power legitimised within modern nation states.

By the second half of the 20th century, the focus had shifted from a vision of the social contract as necessary to ensure security to a more progressive approach which gave greater consideration to the active role of citizens in shaping the society in which they live. The *new* social contract envisaged by former French Prime Minister and Minister of Education Edgar Faure at the beginning of the 1970s (e.g. Faure 1973) was the result of his seminal work as politician and intellectual. Faure was asked to chair the International Commission on the Development of Education set up by UNESCO in 1971, and its final report, entitled *Learning to be: The world of education today and tomorrow,* is commonly known as the Faure report (Faure et al. 1972). Although not explicitly mentioned in the report, this idea of a new social contract constituted the basis of the analysis and reflections on the urgency to radically rethink learning systems from the perspective of what was referred to as “lifelong education” (translated from the French term *éducation permanente*). Faure’s new social contract had clear philosophical and political contours: it was based on a strong humanistic perspective, which was at odds with more instrumental approaches to education based on human capital theory, and portrayed an alternative vision on how political systems should be organised to ensure equity and social justice. In this sense, the new social contract seemed to provide a viable political framework for re-establishing the particular relationship between education and society, based on a strong belief in an educational democracy which considered citizens as real agents of change.

Today, fifty years after the publication of the Faure report, the challenges that education systems are facing worldwide do not appear to have changed substantially. Nevertheless, they have gained momentum. Environmental problems, technological acceleration and its impact on human conditions and on the rapidly changing world of work, the need to tackle the long-term effects of colonisation on education systems, and the crisis of education, already addressed in the Faure report, were revisited 24 years later in *Learning: The treasure within* (Delors et al. 1996), commonly known as the Delors report, and they continue to challenge us.

The onset of the current pandemic greatly increased our awareness of the interconnectedness and interdependence among people worldwide, but also highlighted both new and existing inequalities. This awareness has led international organisations, youth and civil society movements to call for social, economic and political renewal in order to revisit the policies and norms that govern how we live together in a society. What we now need is referred to by United Nations Secretary-General António Guterres as “a new social contract for a new era” (Guterres 2020).

Another commission set up by UNESCO more recently, in 2019, is the International Commission on the Futures of Education (ICFE), chaired by Sahle-Work Zewde, President of the Federal Democratic Republic of Ethiopia. Its report, *Reimagining our futures together: A new social contract for education* (ICFE 2021) takes up the idea of a new social contract as a framework to address the issues affecting education worldwide. Published in November 2021, this report asks the question: What would a different social contract for education better suited to the needs of the 21st century look like? According to the vision set out in this report, a new social contract *for education* is considered essential for reimagining our futures, for repairing past injustices and building a more equitable and sustainable planet (ibid.).

This article provides a review of Faure’s new social contract and its influence on the Faure report (Faure et al. 1972), and compares it with the vision elaborated within UNESCO’s more recently published *Futures of Education* report (ICFE 2021). While the understanding of the social contract elaborated by Faure had clear political contours, as already mentioned, and translated into an up-to-date vision of the emancipatory function of education for the fulfilment of individuals within democratic societies, its definition was vague and the influence of the Faure report in the elaboration of new education policies based on the concepts of “lifelong education” and a “learning society” remained limited. By comparison, in advocating for a new social contract for education, the *Futures of Education* report adopts a more communitarian (and environmentally conscious) perspective based on the idea of *togetherness*.

Acting *together* is seen as both a means and an end to the way in which the social contract should be re-established in its new, updated form. This represents a significant shift with regard to the report’s target readership. Indeed, while both the Faure and Delors reports (Faure et al. 1972; Delors et al. 1996) were addressed to governments as main implementers and referred to the nation-state as the unit that guides the development of education, the *Futures of Education* report addresses “*all* actors” (ICFE 2021, p. 139; emphasis added) involved in education at *all* levels. This portrays a different vision of the nature of the State and its changing role in the governance of education at both national and global levels. However, in this report, the definition of the new social contract for education is also vague and the political discussion on the relationship among the institutions that should govern it is less explicit than in the Faure report.

This article discusses the extent to which the principles underpinning a new social contract for education, especially the notion of education as a common good, provide the political framing of such a new social contract for education. It ultimately examines the relevance of the political discussion of the relationship between education and society elaborated in the Faure report fifty years ago with regard to the formulation of a new social contract for education for the twenty-first century.

## The conceptualisation of the new social contract in the work of Edgar Faure: implications for the *Learning to be* report and its influence on the two subsequent commissioned UNESCO reports

Faure first presented his notion of a new social contract in a pamphlet published by *Le Monde* in 1963.^1^ His idea represented an evolution of the social contract as elaborated by Jean-Jacques Rousseau. According to Faure,

the first social contract was a contract of security. The second is a contract of progress (Faure 1973, p. 90; my translation).

The latter goes beyond the initial concept of a democracy based on a legal agreement and combines it with the idea of a *beneficiary democracy,* which establishes a different relationship between the State and the citizen. As indicated in the 1963 pamphlet,

in addition to the representation of the individual citizen, it will be necessary to organise a representation of the same citizen as an agent of the different sectors of the economy (Faure 1973, p. 96, my translation).

The new social contract elaborated during the 1960s, and re-presented at the beginning of the 1970s, was related to the idea that justice and progress are two interrelated facets of the social contract, and that economic development cannot be achieved without proper concern for the development of individuals as main agents of social progress. As elaborated by Faure in 1963,

[w]e are now on a clearly defined path, which is neither that of clear liberalism nor that of proper socialism. [...] it involves an equitable distribution of the (disposable) national income, a “functionalisation” of the income movement (Faure 1973, p. 95, my translation).

In this regard, the new social contract conceptualised by Faure “was situated in the Keynesian democratic welfare state, the dominant economic model at the time”, and was considered a means of “controlling capitalism”, by “spending its surplus for the benefit of those in need” (Elfert and Morris 2022). Faure’s idea was clear: true democracy, in a context of societal and economic progress, required a reorganisation of the State and a combination of classical liberalism and socialism.

The understanding of the social contract called for by Faure represented an attempt to go beyond the classical social contract theory as developed by Hobbes, Locke and Rousseau, which referred to the process of state formation and was “merely the precondition for living in a society free from exploitation” (Shafik 2021, p. 6). This contract was based on a transactional agreement among rational individuals seen as sovereign subjects within a vision of society instrumental to the preservation of individual rights and benefits (Toukan 2021). It could be argued that Faure’s adoption of a more progressive interpretation of this contract was in line with recent developments of social contract theory, which gave greater relevance to the principles of distributive justice, human rights and human capabilities and laid greater emphasis on solidarity and cooperation among individuals.^2^

The concept of the *new* social contract elaborated by Faure was related to the idea of a *beneficiary* democracy which required citizens to be not only passive citizens but real agents of change. The new social contract therefore appears to be interrelated with the humanistic approach developed by the International Commission on the Development of Education under the chairmanship of Faure, who was extremely critical towards more utilitarian and technocratic approaches to education (Elfert 2018). Indeed, Faure, who considered himself a humanist, had a considerable influence on the work of the International Commission convened in 1971 by René Maheu, then UNESCO Director-General, to identify new strategies for the future of education and of mankind in relation to the “crisis of education” that was apparent at the threshold of the Second United Nations Development Decade (UNESCO 1971). Faure called into question the very notion of “capital” within human capital theories and underlined that

the danger represented by the technocratic and economic approach required the emergence of a “new man” endowed with the capacity to understand the world in which he/she lives, to take decisions and maintain autonomy that would allow him or her to resist the instrumentalisation inherent in the development of society (Elfert 2018, p. 121, referring to Faure et al. 1972).

In this regard, the essential purpose of education, as expressed in the title of the report *Learning to be*, indicates the full development of individual citizens as the necessary condition for the effective functioning of modern democracies. As argued by Asher Deleon, “[t]he Report focused on personal development and put learners, not teachers or educational institutions, at the core of education” (Deleon 1996, p. 13).

The reflections developed in the Faure report can be considered anticipatory of two subsequent UNESCO flagship reports on learning and education: the Delors report (Delors et al. 1996) and more recently the *Futures of Education* report (ICFE 2021). As mentioned above, the tensions and challenges identified at the beginning of the 1970s are still relevant, although they have changed in nature and scope. For instance, the Faure Commission warned against the potentially harmful effects of uncontrolled scientific and technological progress on the environment and on human beings. The Preamble of the Faure report focuses on the risks of de-humanisation resulting from the acceleration of technological changes, “affecting privileged and oppressed alike” (Faure et al. 1972, p. xxi). According to the Faure Commission, the only way out of this negative scenario was through democracy, humanistic development and change, hence the crucial role of education in “bringing out the full potential of human beings and enabling them to shape their societies towards greater democratization and social justice” (Elfert 2018, p. 1). The need for a humanistic approach to education was reaffirmed in the Delors report (Delors et al. 1996), where humanism considered the relationship among human beings, in contrast to the more market-oriented approach to education which was prevalent in the 1990s (Tawil and Cougoureux 2013).

More recently, the *Futures of Education* report (ICFE 2021) not only reaffirms the humanistic approach of both the Faure and the Delors reports, but goes even further and includes reflections on the relationship between human beings and the natural environment. It could be argued that the humanistic approach called for in the latest UNESCO report (ICFE 2021) appears to be based on a clear collective and cooperative dimension of education, while “the humanism of the Faure report is individualistic *before* it becomes collective and political” (Biesta 2021, p. 5, emphasis in original). This is expressed in the Faure report’s call for a new social contract for education, which should be based on a vision of education as a shared societal endeavour. In this regard, while maintaining continuity with the two previous UNESCO reports (Faure et al. 1972; Delors et al. 1996), the *Futures of Education* report (ICFE 2021) distinguishes itself by addressing not only governments, but *everyone* involved in education, thus reflecting more recent developments in the governance of education in increasingly complex systems.

## An increasing call for a new social contract, also in education

In the last couple of years, the pandemic revealed how limited the capacities of traditional welfare states may be to ensurethe distribution/enjoyment of collective benefits, which can only be achieved through a shared commitment by society’s different constituencies. Many of today’s challenges, further emphasised by the pandemic, call for a revision of the norms and rules governing how collective institutions operate, i.e. the “social contract”. On Nelson Mandela International Day in July 2020, in the aftermath of the first wave of the coronavirus pandemic, António Guterres called for “a new social contract” to address the injustices caused by the pandemic (Guterres 2020). Based on this lecture, the UN Secretary-General published a report in September 2021, entitled *Our Common Agenda*, which acknowledges that

*now is the time to renew the social contract between Governments and their people and within societies,* so as to rebuild trust and embrace a comprehensive vision of human rights (UN 2021, p. 4, emphasis in original).

Guterres refers to the need for a new social contract at the national level, which should be complemented by a “new global deal” to ensure a deeper commitment to international solidarity. This is considered necessary for delivering global public goods and protecting global commons (UN 2021).

The report clarifies that while the term “social contract” has its origins in Western or European philosophy, similar concepts – which reflect “reciprocal obligations between people, households, communities and their leaders” (ibid., p. 22) – can be found across different religious traditions and regions worldwide. Even though there is no precise definition of the term “social contract”, which according to the report “originates at […] national and subnational levels” (ibid.), its foundations are identified in the following principles: “*(a) trust; (b) inclusion, protection and participation; and (c) measuring and valuing what matters to people and the planet*” (ibid., emphasis in original). As recalled by Guterres in his lecture (Guterres 2020), the new social contract is necessary to counter a vision of the economy which has produced ever-increasing inequality worldwide. However, as argued by Elena Toukan, “the notion of the new social contract identified here continues to be transactional and utilitarian in nature, describing cooperation primarily as a means for exchange and solutions” (Toukan 2021, p. 6).

Recently, reference to the social contract concept has also been made in the field of education. While education can be considered as an essential component of the social contract *per se*, it has at the same time been seen as a social contract itself. It is this latter point of view that has been adopted in UNESCO’s recent report on the Futures of Education:

Education can be seen in terms of a social contract – an implicit agreement among members of a society to cooperate for shared benefit. A social contract is more than a transaction as it reflects norms, commitments and principles that are formally legislated as well as culturally embedded (ICFE 2021, p. 2).

The way in which the social contract is understood in the latest UNESCO report (ICFE 2021) to a certain extent reflects the way in which it is conceptualised in the UN Secretary-General’s report, *Our Common Agenda* (UN 2021). The *Futures of Education* report, however, goes even further by suggesting a paradigm shift from a merely transactional to a relational vision of society, and therefore of the social contract (Toukan 2021).

According to ICFE, the need to re-establish a new social contract for education is based on the acknowledgement that the social contract for education of the 19th and 20th centuries should be revised. This would involve taking into account what has worked, what needs to be abandoned, and what needs to be reimagined so as not to reinforce existing power structures which would continue to lead to marginalisation and to the reproduction of inequalities. ICFE suggests that a new social contract for education should favour a profound revision of the “principles for organizing learning” in the areas of pedagogy, curriculum, teaching, schooling and education systems’ organisation (ibid, pp. 46–47). In order to overcome the limitations of the existing schooling system (in the United States, but by inference also elsewhere), Jal Mehta also calls for a new social contract for learners, “a new grammar of schooling” and a new social commitment for learning communities (Mehta 2022). The need for a new grammar of schooling became more evident during the height of the pandemic, which highlighted the limited means of existing schooling systems and of public authorities to sustain education opportunities, which were supplemented by the capacity of resilience, creativity and innovation that existed in ordinary people.^3^

As mentioned in the *Futures of Education* report,

a range of governmental and non-state partners need to work together to meet unfulfilled commitments of the past and unlock the transformative potential of education for the future (ICFE 2021, p. 119).

This is the reason why this most recent report, as well as being addressed to everyone involved in education, also emphasises the need for a bottom-up approach on how to transform education for the future, a vision which was less evident in the two previous UNESCO reports (Faure et al. 1972; Delors et al. 1996). The nature of the new social contract for education outlined in the *Futures of Education* report is reflected in the principles which should govern it, namely (1) “an expanded vision of the right to education throughout life”, which includes the right to information, science, culture, and the right to participation; and (2) the concept of education as a public shared endeavour and a common good (ICFE 2021, p. 146).

## Strengths and weaknesses of the new social contract for education^4^

While acknowledging the remarkable contribution of the *Futures of Education* report, critiques have been raised with regard to the vagueness of the notion of the “new social contract for education” (Elfert and Morris 2022; Klees 2022; Tarozzi and Milana 2022). In relation to previous UNESCO reports, the latter “does not assign the same urgency to democracy as the *Faure report* did” and avoids addressing the political stance inherent to the notion of the social contract (Elfert and Morris 2022, p. 41; italics in original). It has been argued that “[t]he report lacks a critical analysis of the structural obstacles to the implementation of the ideas it presents, in particular it lacks an analysis of power” (ibid.). Moreover, the report does not fully engage with the issue of the privatisation of education, which is “one of the chief challenges we face in these neoliberal decades” (Klees 2022). In this section, I investigate the challenges to the realisation of the new social contract called for by Faure and its contribution to the political discussion around the governance of education, and compare it with the vision presented in the *Futures of Education* report.

It has been argued that the ideas and concepts presented in *Learning to be* (Faure et al. 1972) had no significant influence on national education policies (Elfert 2022). This is mainly attributed to the spread of a narrow vision of lifelong learning as a result of the influence of neoliberal market-oriented policies in the late 1970s, which were opposed to the egalitarian nature of lifelong learning and to the active participation in society that the idea of lifelong education entailed (Elfert 2022). Moreover, the Faure report contained an ideological critique of the traditional schooling model, which raised some attention and made the report controversial. While the research programme engaged in by the UNESCO Institute for Education (UIE) in the 1970s following the publication of the Faure report employed the discourse on non-formal and informal learning, it was too focused on pedagogical and curriculum aspects of primary and secondary education and did not address issues more related to adult education (Elfert 2022).

The same observation can be made regarding the *Futures of Education* report. While this publication “includes calls for a broad, multidimensional and transformative view of adult education”, “this vision will likely remain at the level of rhetoric given that […] adult education has not made its way into the indicators of SDG 4” (Elfert 2022, p. 2).^5^ By contrast, what the envisaged expansion of the social contract relies on is also the acknowledgment of the value and contributions of informal and non-formal approaches to learning which are necessary for the evolution of the welfare state and the reduction of inequalities.

Other considerations which should also be taken into account concern the governance foreseen within the new social contract envisioned both by Faure and in the *Futures of Education* report. The emphasis in both the Faure and the *Futures of Education* reports on the need for greater cooperation in the sphere of education may be seen as a response to the particular events of the respective times in which the two reports were drafted. The former was prepared just after the 1968 students’ uprisings, and the latter in the wake of youth and civil society movements for climate and social justice, and during the Covid-19 pandemic. All of these events have emphasised the need to radically rethink education systems on the basis of more democratic and participatory principles. Indeed, in a recent article on the distinctiveness of the *Futures of Education* report in its framing and generation of different educational futures compared to previous UNESCO global reports, Noah W. Sobe argues that there are more similarities between the Faure and the *Futures of Education* reports compared to the Delors report, as they both call into question the “linear expansion of education” (Sobe 2022, p. 4).

Having said this, it is worth noting that while highlighting the humanistic vision of learning and of society, the new social contract called for by Faure remained mainly framed within a transactional individualistic perspective. In education, the main focus of the Faure report was on learners seen as real agents of change, but this did not imply the assumption of a different ontological vision of society, as the *Futures of Education* report appears to suggest instead. Indeed, the latter highlights relationality versus transaction in the formulation of a new social contract which relies on the intrinsic collective and interconnected dimension of society (Toukan 2021). It could however be argued that the Commission chaired by Faure put forward a clear political stance for democracy and education, underlining the necessary role that a welfare state should play in the governance of education.

During the press conference which launched the *Learning to be* report in 1972 (UNESCO 1972), one journalist asked whether the strategies mentioned in the report were to be applied in a neutral or blank ideological terrain. Faure replied to that provocation by asserting that the theories developed by his Commission could be applied in any ideological/political domain on condition that it was characterised by the quest for real democracy in education (ibid.). Majid Rahnema, one of the commissioners, added that the political character of education had been widely emphasised in the report and, although the Faure Commission had not specified which political structures were supposed to be more favourable for human development, it had made clear that these conditions depended on a strong political will (ibid.).

The reaffirmation of the public character of education is also present in the *Futures of Education* report (ICFE 2021), but its political analysis remains more implicit than in the Faure report (Faure et al. 1972). ICFE presented a vision of the possibilities of imagining alternative futures in education, but the discussion on the conditions and on the relations of power that prevent or favour the realisation of these futures are less evident. Such a reflection is all the more urgent considering the rising challenges to democratic institutions, the increasingly complex governance of education and the trends towards the commercialisation and privatisation of education at all levels and dimensions, with potential impact on the rise of inequality. In particular, recent developments and concerns on the growing involvement of non-state actors in education have called into question the traditional role of welfare states in the provision of collective goods, such as education. It follows that, especially in education, the issue of what the role of the State should be within the social contract remains fundamental and raises important political questions, especially when advocating for society-wide commitment, as the *Futures of Education* report suggests.

## Relevance for the concept of education as a common good

This last section asks whether the concept of education as a common good may help to clarify the political implications resulting from the new social contract for education from the point of view of education governance. The need for greater cooperation in the educational endeavour was already mentioned in the Faure report (Faure et al. 1972), which underlined the possible benefits of shared governance, but also identified the limits thereof. In fact, it recommended considering two key political questions in education reforms:

First, who among participants, users and others concerned should share this right to guide and manage education, as a joint enterprise? […] Second, at exactly what level should co-management and self-management operate, and to what extent? (Faure et al. 1972, p. 78).

In this sense, Faure and the members of his Commission raised important questions about legitimacy when dealing with the sharing of educational responsibilities, regarding who, among the different actors, should be entitled to take part in the formulation of education policies and be officially recognised by governments in this process.

As mentioned above, rather than raising broader political questions on critical issues that may influence the democratic governance of education, the *Futures of Education* report (ICFE 2021) underlines the *method* around which the new social contract should be built, i.e. by means of dialogue involving the widest possible participation, as expressed through the use of the term *together*. The idea of *commonness* or *togetherness* in education, which is one of the main features of the *Futures of Education* report (ibid.), was already pronounced in the Delors report (Delors et al. 1996). Indeed, the International Commission on Education for the Twenty-first Century, chaired by Jacques Delors, emphasised that “learning to live together” would be crucial for future education in light of globalisation trends and the increasing frequency of interaction among peoples of different civilisations and identities on the threshold of the 21st century. The *Futures of Education* report strengthens this idea, identifying the principle of “education as a public endeavour and a common good” (ICFE 2021, p. 2) as one of the foundations for governing the new social contract for education:

As a shared societal endeavour, education builds common purposes and enables individuals and communities to flourish together. A new social contract for education must not only ensure public funding for education, but also include a society-wide commitment to include everyone in public discussions about education. This emphasis on participation is what strengthens education as a common good – a form of shared well-being that is chosen and achieved together (ibid.).

The adoption of the concept of “education as a common good” as one of the principles governing the new social contract for education reflects the increasingly complex governance of education at different levels. Having said this, the way in which this concept is defined seems to refer to the more philosophical concept of *the* common good as such, rather than to a principle of education governance. These elements deserve greater attention, since the category of “common goods” (ibid., p. vi) has a strong political connotation and may provide useful elements for the discussion on the governance of education within the new social contract.

Common goods are considered necessary for the realisation of fundamental human rights (Rodotà 2013) and their declaration has been identified as a reaction to the subjection of public goods to market logics (Fidone 2017, 2021). However, unlike public goods, which can be enjoyed as individual goods, common goods are considered as relational goods and therefore presuppose forms of shared governance for both their production and enjoyment (UNESCO 2015). For instance, education can be considered as a *public* good in its traditional form of instruction which is guaranteed publicly, but can always be enjoyed individually. Education as a *common* good instead requires all components of society to take responsibility in the education process, and the results can only be enjoyed collectively (see for instance the experiences of community or territorial education pacts, service-learning initiatives, open schools movements).^6^

The concept of *education as a common good* requires, and at the same time reinforces, active citizenship, equality, and community empowerment, which represent the preconditions for effective participation in the democratic process. This manifests itself as a clear political perspective which is grounded in a strong sense of solidarity among the different components of society and translates into organisational structures which highlight inclusion, cooperation and solidarity at different levels, ranging from policymaking, decision-making, leadership, and governance to implementation and practice. Seeing education as a common good therefore necessarily implies an integrated approach which gives the same value to all forms and levels of education and learning and may favour the transformation of public institutions in order to overcome more hierarchical and utilitarian approaches and build more democratic schooling systems. It requires students, families, communities and other actors to be prepared, to acquire the capabilities needed to participate freely and responsibly in the educational process, and therefore to contribute to the strengthening of democratic institutions. This is necessary to effect a shift from *formal* democracy, which is mainly an “aggregative technique” and limits itself to representation, to *participatory* democracy, which is the most effective way for society to put forward its visions of well-being (Locatelli 2019).

In this regard, within the framework of education as a common good, the possibility of an open dialogue on the aims and organisation of education ultimately relies on the existence of a democratic system that can realistically be guaranteed by the State, and this is crucial when renewing the social contract for education. It is the premise for any action taken in view of the development of more inclusive and participatory systems, which was evident in Faure’s vision of the new social contract and in the *Learning to be* report (Faure et al. 1972).

## Conclusions

In order to address the long-standing crisis in education and significantly reshape the way in which public institutions themselves function, there is a need for a shift in culture – a transformative change, combining top-down and bottom-up approaches. These changes need to be substantial and “cannot be reduced to mere adjustments in a machine which has lost sight of its own purpose” (Tedesco 1995, p. 107). This concern was present in the Faure report five decades ago when it stated that for democracy to be

living, creative and evolving […] social structures must be changed […] Educational structures must be remodelled, to extend widely the field of choice and enable people to follow lifelong education patterns (Faure et al. 1972, p. 79).

This emancipatory vision of education was linked to the idea of a new social contract which highlighted cooperation, but remained anchored to an individualistic, albeit humanistic, understanding of society.

Over the past fifty years, the call for a new social contract has become a call for greater concertation among societal actors. In order to radically rethink education systems, address past injustices and build more sustainable futures, the new social contract for education called for in the latest UNESCO report (ICFE 2021) suggests a paradigm shift from a transactional to a relational model. Hence, while the Faure Commission maintained a more government-centric approach and identified cooperation as one of the ways of promoting educational reforms (Faure et al. 1972), the *Futures of Education* report takes this even further: acting *together* is seen as the best method to think of and achieve new educational futures (ICFE 2021), a term intentionally used in the plural form.^7^ In this sense, the *Futures of Education* report reflects and extends the attention paid in the Faure report to the emancipation of education, to the possibility of “keeping the future ‘open’ for the new generation rather than determining their future for them” (Biesta 2021, p. 12).

The understanding of education which is at the basis of the new social contract called for in the most recent UNESCO report (ICFE 2021) counters a vision of education seen mainly as an individual, albeit emancipatory, matter. It is built on the assumption that while change depends on a strong political will, it is also shaped/determined by the capacity of citizens, political and social groups to put pressure and bring attention to particular issues. The concept of education as a common good is indeed based on the capacity of human beings to take responsibility for their own actions and, at the same time, on the belief that educational change can only be achieved *together*.

International reports are of course products of the times in which they are written, and the concept of the social contract will probably continue to undergo new interpretations in the future. Despite its limitations, the claim made by Faure regarding the need of a political discussion on the nature of education and its relationship to society and democracy, is now more relevant than ever. The new social contract for education called for in the *Futures of Education* report (ICFE 2021) allows for a reaffirmation of the relational dimension of education. However, the conditions for its realisation vary considerably within different countries and societies. For the new social contract for education to be truly transformative, a profound discussion on how shared governance can be fulfilled and how responsibilities should be distributed within different futures has to follow.

## Data Availability

Data sharing not applicable to this article as no datasets were generated or analysed during the current study.

## Footnotes

1 The full pamphlet « Le nouveau contrat social » was included in the volume *Pour un nouveau contrat social* (Faure 1973).

2 This interpretation reflects the critical reviews of the social contract as developed by John Rawls (1971), Charles R. Beitz (1999 [1979]) and more recently by Samuel Moyn (2013). Minouche Shafik points out that political economist Adam Smith (1723–1790) already talked about the need of “circles of sympathy” as the foundations for modern economics, referring to self-interested individuals who also cared about the well-being of others (Shafik 2021, p. 6).

3 These ideas were expressed by Fernando Reimers, member of the International Commission on the Futures of Education, during the online seminar *Lucca Learning City Unesco: Re-immaginare il futuro dell'educazione insieme* (Reimers 2022).

4 I am grateful to the anonymous reviewers for the accurate comments that made it possible to enrich and strengthen this section.

5 For details of the indicators of the fourth Sustainable Development Goal (SDG 4) within the United Nations 2030 Agenda, see UIS (2018).

6 *Educational pacts* are specific agreements between school institutions and local educational agencies (public training bodies, museums, associations) drawn up for the purpose of enriching the educational activities of schools and actively involving the community. The purpose of *service-learning initiatives* is to provide students with opportunities for experiential learning through volunteering e.g. with a non-profit organisation. *Open school initiatives* relate to the opening up of the school after school hours to the neighbourhood, involving students/former students, parents, local citizens and third-sector entities as co-organisers.

7 According to its dedicated website, the Futures of Education initiative “uses the concept of futures in the plural in order to recognize that there is a rich diversity of ways of knowing and being around the world. The plural form also acknowledges that there are multiple dimensions to the future and that there will likely be various desirable and undesirable futures – all of which will vary greatly depending on who you are and where you stand. Rather than attempting to chart a single future, looking at futures in the plural validates multiple possible and desirable futures of humanity on our shared planet” (UNESCO 2019).
